# Anakinra authorized to treat severe coronavirus disease 2019; Sepsis breakthrough or time to reflect?

**DOI:** 10.3389/fmicb.2023.1250483

**Published:** 2023-10-19

**Authors:** Leland Shapiro, Sias Scherger, Carlos Franco-Paredes, Amal Gharamti, Andrés F. Henao-Martinez

**Affiliations:** ^1^Division of Infectious Diseases, Department of Medicine, Rocky Mountain Regional Veterans Affairs Medical Center, Aurora, CO, United States; ^2^Division of Infectious Diseases, Department of Medicine, University of Colorado Anschutz Medical Campus, Aurora, CO, United States; ^3^Hospital Infantil de México, Federico Gómez, México City, México; ^4^Department of Microbiology, Immunology, and Pathology, Colorado State University, Collins, CO, United States; ^5^Department of Internal Medicine, Yale University, Waterbury, CT, United States

**Keywords:** anakinra, COVID-19, sepsis, cytokines, cytokine storm, inflammation, Interleuhin-1, Interleukin-1 receptor antagonist

## Abstract

**Introduction:**

The European Medicines Agency (EMA) and the United States Food and Drug Administration (FDA) announced conditions for using recombinant human interleukin-1 receptor antagonist (rhIL-1ra) to treat hospitalized patients with Coronavirus disease 2019 (COVID-19) and risk for progression. These decisions followed publication of the suPAR-guided Anakinra treatment for Validation of the risk and early Management OF seveRE respiratory failure by COVID-19 (SAVE- MORE) phase 3 clinical trial that yielded positive results.

**Methods:**

We conducted a literature review and theoretical analysis of IL-1 blockade as a therapy to treat COVID-19. Using a stepwise analysis, we assessed clinical applicability of the SAVE-MORE results and evaluated conceptual support for interleukin-1 suppression as a suitable approach to COVID-19 treatment. This therapeutic approach was then examined as an example of inflammation-suppressing measures used to treat sepsis.

**Results:**

Anakinra use as a COVID-19 therapy seems to rely on a view of pathogenesis that incorrectly reflects human disease. Since COVID-19 is an example of sepsis, COVID-19 benefit due to anti-inflammatory therapy contradicts an extensive history of unsuccessful clinical study. Repurposing rhIL-1ra to treat COVID-19 appears to exemplify a cycle followed by inflammation-suppressing sepsis treatments. A landscape of treatment failures is interrupted by a successful clinical trial. However, subsequent confirmatory study fails to replicate the positive data.

**Discussion:**

We suggest further experimentation is not a promising pathway to discover game-changing sepsis therapies. A different kind of approach may be necessary.

## Introduction

Anakinra (recombinant human interleukin-1 receptor antagonist or rhIL-1ra) was fully approved by the European Medicines Agency (EMA) human medicines committee for use in Coronavirus Disease 2019 (COVID-19) that followed a prior indication extension 17 December 2021. In the United States an emergency use authorization (EUA) from the Food and Drug Administration (FDA) was issued for anakinra to treat COVID-19 on 8 November 2022. Patient eligibility criteria include hospitalized adults infected with Severe Acute Respiratory Syndrome Coronavirus Type 2 (SARS-Cov-2) with pneumonia requiring oxygen supplementation and risk for progression. Progression risk is defined by increased inflammation, presumedly indicated by elevated soluble urokinase plasminogen-activator receptor (suPAR) levels, or by a list of patient characteristics issued by the FDA (see below). The phase 3 clinical trial SAVE-MORE (suPAR-guided Anakinra treatment for Validation of the risk and early Management OF seveRE respiratory failure by COVID-19) provided substantial rationale for endorsing rhIL-1ra for clinical use for treatment of COVID-19 (Kyriazopoulou et al., 2021b). Interest in treating COVID-19 with rhIL-1ra represents the latest chapter in a recurring story. We refer to the use of anti-inflammation strategies designed to treat serious infections (severe sepsis). This approach has followed a pattern consisting of extended periods of negative clinical trial results punctuated by an occasional positive study. In every case, the impact of positive studies weakens over time and fails to deliver accepted sepsis therapies. The recent authorizations for anakinra to treat COVID-19 motivated us to determine whether we are entering another such cycle that will terminate in disappointment. On the other hand, is it possible this case is different and COVID-19 sepsis is treatable with anakinra anticytokine therapy? Recent acceptance of anakinra to treat COVID-19 provides a rare opportunity to predict the subsequent fate of this clinical application before a final determination of efficacy (or not) is established. As presented in this hypothesis and theory report, we believe there will be trouble ahead leading to declining interest in this approach to COVID-19 treatment. If our prediction proves correct, this will provide firmer evidence our analysis has merit than if we describe events retrospectively. We accept the risk that our prediction may prove incorrect. Anakinra is a prototype specific cytokine (interleukin-1 or IL-1) antagonist designed to quell the so-called cytokine storm thought to cause sepsis. As such, we evaluate existing experimental and conceptual information supporting or questioning anakinra use in COVID-19. We view anakinra as a case study for prospective assessment of our theoretical concerns about the hyperinflammation approach to sepsis.

Our analysis is segregated into 4 steps. In **Step 1** we present evidence supporting anakinra use for COVID-19. A description of the SAVE-MORE study is presented, and it is shown to possess many laudable design characteristics. In **Step 1a** we explain the motivation to develop non-direct acting agents like anakinra to treat COVID-19 and other causes of sepsis. In **Step 2** we describe reasons for caution before accepting anakinra as a COVID-19 treatment. This section is divided into **Steps 2a-2e**. In **Step 2a** we describe features intrinsic to SAVE-MORE that pose challenges for using anakinra as COVID-19 therapy. **Steps 2b-e** review the prior experience treating COVID-19 and other causes of sepsis with IL-1 suppressing drugs. Also, interleukin-1 concentrations in COVID-19 and in sepsis are evaluated to determine if amounts are sufficient to cause organ malfunction or death. Analyses in **Step 2** suggest the case for anakinra to treat COVID-19 is weak. In **Step 3** we briefly review our previous detailed explanation why hyperinflammation does not cause sepsis (Shapiro et al., 2022). This section broadens the scope of analysis to include the program of defeating sepsis (including COVID-19) with inflammation-suppressing measures. Finally, in **Step 4** we broaden our scope further and point out the cytokine storm concept has fed myriad potential sepsis therapies into a cycle characterized by a landscape of failures punctuated by an occasional positive clinical study. The occasional positive result never translates to an accepted sepsis treatment.

We conclude there is no special reason to believe anakinra use to treat COVID-19 will avoid the disappointing pattern of prior cytokine suppressive therapies. We anticipate waning enthusiasm for using anakinra as a COVID-19 treatment. Equipped with an understanding of why we keep failing suggests a qualitatively different kind of research program is needed to discover successful specific sepsis therapies.

### Step 1– the case for recombinant human interleukin-1 receptor antagonist (rhIL-1ra) to treat COVID-19

A pivotal factor supporting use of IL-1ra in COVID-19 is results from the phase 3 SAVE-MORE clinical trial conducted in 37 sites (29 Greece and 8 Italy) (Kyriazopoulou et al., 2021b). This phase 3, randomized, double-blind, placebo-controlled study with intention to treat design enrolled COVID-19 patients between 23 December 2020 and 31 March 2021. This study recruited hospitalized adult male or female COVID-19 patients with molecular test-confirmed SARS-CoV-2 infection, imaging showing lower respiratory involvement, and plasma soluble urokinase plasminogen receptor (suPAR) concentrations ≥6 ng/mL. Exclusions included patients using mechanical ventilation, use of continuous positive airway pressure (CPAP)/bilevel positive airway pressure (BiPAP), or partial pressure of oxygen divided by fraction of inspired oxygen (PaO2/FiO2) <150. Other exclusions included primary immunodeficiency, peripheral blood neutrophil concentration < 1,500/mm^3^, prednisone use ≥0.4 mg/kg/day for >15 days, or use of anticytokine therapy in the previous 30 days. Entry criteria enrolled patents with non-critical severe illness with lower ventilatory tract disease that warranted hospital admission with and with supposed risk for future excessive inflammation predicted by elevated suPAR. Patients were randomized 2:1 (Anakinra 100 mg in 0.67 mL subcutaneous daily for 7–10 days: saline placebo 0.67 mL subcutaneous daily). Patients with severe disease according to WHO definition received dexamethasone 6 mg/day for 10 days as standard of care. Primary outcome was distribution of patients after 28 days within the 11-point World Health Organization (WHO) Clinical Progression Scale (CPS) (Characterisation, WHOWGotC, and Management of, C-i.A, 2020). The score used in this study comprised:

1. Fully recovered and polymerase-chain reaction (PCR) negative for SARS-CoV-2 genome.
2. Asymptomatic with SARS-CoV-2 PCR positive.
3. Symptomatic and functionally independent.
4. Symptomatic and assistance needed.
5. Hospitalized without exogenous oxygen.
6. Hospitalized with exogenous oxygen (nasal cannula or mask),
7. Requirement for high-flow oxygen or non-invasive ventilation (NIV).
8. Mechanical ventilation and paO_2_/FiO_2_ > 150 mmHg.
9. Mechanical ventilation and paO_2_/FiO_2_ < 150 mmHg or vasopressors.
10. Mechanical ventilation and paO_2_/FiO_2_ < 150 mmHg and vasopressor or hemodialysis or extra-corporeal membrane oxygenation (ECMO).
11. Death.

Secondary endpoints included changes in WHO-CPS at day 14 or at day 28 compared to score at enrollment, change in Sequential Organ Failure Assessment (SOFA) score at day 7 vs. enrollment, time to hospital discharge, and time residing in an intensive care unit (ICU) for patients admitted to the ICU. In patients receiving Anakinra (N = 189) or placebo (N = 405), dexamethasone was given at enrollment to 88.4% (Anakinra group) and 88.9% (placebo group). Primary endpoint results showed statistically significant lower 28-day distribution in the WHO-CPS in anakinra patients compared to controls (*p* < 0.001). This included a total 50.4% of anakinra patients with score = 1 compared to 26.5% in control (placebo) group. Statistical analysis indicated treatment effect size was homogenous across all 11 points of the WHO-CPS hierarchy. A mortality difference (not a primary endpoint) favored anakinra patients (score = 11), with 13 deaths in 406 anakinra subjects (mortality 3.2%) compared to 13 deaths in 189 placebo subjects (mortality 6.9%), *p* = 0.045. Median absolute decrease in WHO-CPS score from day 1 to day 28 was numerically larger for anakinra group compared to controls (decrease of 4 compared to decrease of 3 scale points with *p* < 0.0001). Dexamethasone use associated with worse 28-day WHO-CPS distributions scores, and authors ascribe this unexpected result to selective steroid administration to sicker patients. No separate analysis of patients using vs. not using steroids was provided.

A follow-up publication expanded the description of results in SAVE-MORE with a focus on subgroup analyses (Akinosoglou et al., 2023). Anakinra conferred significant WHO-CPS distribution benefit (lower distribution) for COVID-19 patients in all subgroups assessed. Subgroups included enrollees with suPAR levels 6–9 ng/mL or > 9 ng/mL, those of age < 65 or ≥ 65, male compared to female patients, those with Charlson Comorbidity Index <2 or ≥ 2 and benefit regardless of enrolment time after symptom onset (0–7 days, 8–9 days, 10–11 days, or > 11 days). Anakinra benefit defined as lower distribution of WHO-CPS score was maintained at days 60 and 90. Hospital costs were reported to be lower by up to 40% in anakinra patients compared to placebo.

#### Step 1a: what motivates interest in using anti-inflammation strategies to treat COVID-19?

Since high mortalities were associated with severe forms of COVID-19 during early phases of the pandemic, an urgent desire to improve COVID-19 outcomes instigated a search for readily available or “off-the-shelf” therapies (Chakraborty et al., 2021; Govender and Chuturgoon, 2022). In a study reporting on the first 20 months of the pandemic, 104,590 electronic health records were obtained from 21 US health care systems in a retrospective cohort study of hospitalized adult COVID-19 patients (Fiore et al., 2022). A 16.9% standardized mortality February–April 2020 subsequently decreased to 9% later in the pandemic July–September 2021. A Centers for Disease Control and Prevention (CDC) report calculated crude COVID-19 hospital mortalities during the Delta (July–October 2021), early Omicron (January–March 2022), and late Omicron (April–June 2022) periods (Adjei et al., 2022). Decreasing rates of 15.1, 13.1, and 4% were observed during these 3 pandemic periods, respectively. Fall in mortality in these studies likely resulted from improved supportive care, emergence of novel therapies, vaccinations, and virus mutations that produced less lethal SARS-CoV-2 variants. It remains possible mortality may increase in the future due to mutation with emergence of vaccine or therapy-resistant virions or waning immunity.

Motivation to lower COVID-19 mortality applies to mortality in sepsis, since severe COVID-19 is a form of sepsis (Karakike et al., 2021; Kocak Tufan et al., 2021; Vincent, 2021; Herminghaus and Osuchowski, 2022). Sepsis is currently defined as infection with severity sufficient to elevate SOFA score by 2 points (Singer et al., 2016), where SOFA assigns increasing point values for degrees of dysfunction of pulmonary, cardiovascular, hepatic, central neurologic, renal, or coagulation systems. Several sepsis mortality analyses reveal a troubling cessation in mortality improvement. Sepsis mortality was quantified in a meta-analysis of 170 interventional or observational studies conducted 2009–2019 (Bauer et al., 2020). Pooled international sepsis 24- and 90-day mortalities were 24.4 and 32.2%, respectively. For patients with septic shock, 28-day and 90-day mortalities were 34.7 and 38.5%. This report also found a time-dependent decline in 30-day septic shock mortality until 2011 that was followed by cessation of improvement after 2011. A separate study of sepsis 28-day mortality in 44 randomized controlled trials (RCTs) 2002–2016 showed no significant improvement in sepsis mortality between 1991 and 2013 after adjusting for sepsis severity (Luhr et al., 2019). Considered together, mortality assessments in COVID-19 and sepsis indicate there is significant residual mortality despite best medical care. Moreover, it appears progress in sepsis mortality reduction have stalled. Although COVID-19 mortality has shown gratifying reductions, there is no guarantee more lethal variants will not emerge and result in a prolonged period of increased mortality. Residual mortality despite best medical care in COVID-19 and sepsis is the target of adjunctive therapies. Adjunctive treatments are those separate from supportive care and direct acting antipathogen drugs (antibiotics or antivirals). Interventions that suppress host inflammation occupy a central position in the thinking used to devise adjunctive treatments.

### Step 2– the case against anakinra to treat COVID-19

Authorizations to use anakinra to treat severe COVID-19 appear to rely on problematic evidence and rationale. We focus on two different kinds of reservations. The first kind concerns intrinsic qualities of the design of the SAVE-MORE study. The second or extrinsic kind concerns the conceptual foundation that undergirds anakinra COVID-19 therapy; namely using anti-inflammation measures to treat COVID-19.

#### Step 2a: SAVE-MORE intrinsic concerns

European approval and American FDA EUA to use anakinra to treat COVID-19 put forth eligibility criteria based on SAVE-MORE. The FDA EUA applies to hospitalized adults with positive SARS-CoV-2 viral testing and pneumonia requiring supplemental (low or high-flow) oxygen, are at risk of progressing to respiratory failure, and thought likely to have elevated circulating suPAR ≥6 ng/mL. Since suPAR is not a readily available clinical test, the FDA EUA lists surrogate indicators thought to associate with increased baseline suPAR. The EUA stipulates presence of 3 or more of the following 8 characteristics:

1. Age ≥ 75 years.
2. Severe pneumonia by WHO criteria.
3. Current/previous smoking.
4. SOFA score ≥ 3.
5. Neutrophil-to-lymphocyte ratio ≥ 7.
6. Hemoglobin ≤10.5 g/dL.
7. History of ischemic stroke.
8. Blood urea ≥50 mg/dL and/or medical history of renal disease.

The FDA substituted clinical criteria in place of suPAR quantification as an alternative predictor of future inflammation excess. Methods reported in the SAVE-MORE study appear to be strong (Kyriazopoulou et al., 2021b). However, some features of this anakinra COVID-19 trial weaken the case for widespread application to COVID-19 patients. The SAVE-MORE primary endpoint of distribution within the WHO-CPS scale is unusual for a sepsis-related study and authors employ a slightly modified scale compared to the original WHO description (Characterisation, WHOWGotC, and Management of, C-i.A, 2020). Different distributions in WHO-CPS scores between study groups was statistically significant but small in absolute magnitude (median 1 WHO-CPS level improvement compared to baseline in anakinra group compared to controls). Moreover, we find different distributions in the WHO-CPS score challenging to interpret clinically, and this is not unusual for this kind of endpoint. Other than death, patients can transfer up or down the remaining 10 levels of disease severity during the study and presumedly continued to do so after study completion. Since only mortality is unmodifiable, it is possible anakinra causes temporary beneficial WHO-CPS distribution that disappears with time. Eventually all enrolled patients will probably reside in levels 1 (fully recovered and polymerase-chain reaction (PCR) negative for SARS-CoV-2 genome) or 11 (death). The mortality benefit reported in SAVE-MORE (a secondary endpoint) seems likely to represent a fragile result that may diminish over time and lose statistical significance (see below). Skepticism about anakinra effect on mortality derives from experience in prior sepsis phase 3 IL-1ra trials. Two well-designed studies did not demonstrate mortality benefit in a combined study population of 1,589 patients (Fisher et al., 1994; Opal et al., 1997). On the other hand, it is possible patients residing in levels 1–10 may develop prolonged disability due to COVID-19 infection. Perhaps anakinra will lower this risk, but this was not explored in this study. It is hoped SAVE-MORE investigators report longer-term follow up for study subjects that will define the final outcomes of enrolled patients after hospital discharge and illness resolution. Will there be long-lasting anakinra benefit? This will entail follow-up for a longer period than reported so far (Akinosoglou et al., 2023).

The SAVE-MORE primary endpoint deserves comment. It is a kind of composite endpoint since 2 or more outcomes are combined into a single endpoint that is compared between control and intervention groups. Use of composite endpoints has been commented on specifically for use in sepsis or COVID-19 studies (Brown and Ezekowitz, 2017; Desai and Gyawali, 2020; Brown et al., 2021). Composite endpoints are gaining popularity in cardiology studies, are being used more frequently over time, and possess several advantageous qualities. These include increased efficiency since the probability that endpoints accrue during a study is increased compared to lower accrual in studies containing a single endpoint like mortality. This enhances the power of studies to detect significant endpoint differences in treatment arms (Ferreira-Gonzalez et al., 2007a,b, 2008; Armstrong and Westerhout, 2017; Gasparyan et al., 2022). For similar reasons, composite endpoint studies can be conducted faster than other designs, reduce costs and use of other resources, and can capture a more complete range of responses to interventions (McCoy, 2018). These are desirable characteristics for studies conducted during a pandemic where there is urgency to identify useful therapies. The kind of composite endpoint in SAVE-MORE appears to be a variant of a hierarchical composite endpoint (HCE) since outcomes 1–11 are ranked or ordered by severity (Brown and Ezekowitz, 2017; Desai and Gyawali, 2020; Brown et al., 2021; Gasparyan et al., 2022). However, composite endpoints can pose challenges. Composite endpoints can be non-intuitive and difficult to interpret, outcome components cannot be regarded as primary endpoints, disproportionate intervention effect on clinically less important outcomes with smaller effects on more significant outcomes like mortality can distort interpretation of results, and intervention effect on one outcome can be missed due to intervention-induced inhibition of other outcomes (Freemantle et al., 2003; Montori et al., 2005; Ross, 2007; Ferreira-Gonzalez et al., 2007a,b, 2008; Choi and Cheung, 2016; Armstrong and Westerhout, 2017; McCoy, 2018; Palileo-Villanueva and Dans, 2020; Gasparyan et al., 2022; Ramirez and Diaz-Quijano, 2022). A recent positive development in understanding composite outcome results is invention of the maraca plot that graphs HCE results in a single picture (Karpefors et al., 2023). Problems that seem to apply to SAVE-MORE include the presence of a non-intuitive endpoint and outcome components that are of unequal clinical significance (Ferreira-Gonzalez et al., 2007a; McCoy, 2018). Outcomes 1–3 in WHO-CPS are of less clinical significance than outcomes 8–11, making overall anakinra impact on COVID-19 patients challenging to interpret. The primary outcome in SAVE-MORE may prove challenging to translate to patient care and may leave clinicians uncertain what anakinra accomplished in the trial. A thoughtful review of composite endpoints suggests 3 criteria to evaluate the quality of composite endpoints (Montori et al., 2005). The recommended criteria include (1) clinical equivalence (importance to patients) of each outcome, (2) outcome frequencies should be similar in the control group and not favor less clinically-important outcomes, and (3) intervention effects on outcomes should be similar (similar relative risk reductions). The SAVE-MORE study does not satisfy at least the first 2 criteria set forth by Montori et al. (2005).

Mortality or WHO-CPS level 11 was not a primary endpoint in SAVE-MORE. The reported day 28 mortality in anakinra patients was 3.2% (13 of 405), and placebo control mortality was 6.9% (13 of 189 patients, *p* = 0.045). Seven fewer deaths in the placebo group would equalize mortality in placebo and anakinra patients. Furthermore, it is likely fewer than 7 fewer deaths would be needed to convert the value of p to >0.05. The observed SAVE-MORE mortality difference does not appear to be robust. For a study demonstrating mortality benefit with intervention, the fragility index is a mathematical tool that determines the change in number of deaths required to nullify statistical significance of benefit. It is a measure of robustness of trial results (Walsh et al., 2014; Tignanelli and Napolitano, 2019; Andrade, 2020; Dettori and Norvell, 2020; Itaya et al., 2022; Garcia et al., 2023). The SAVE-MORE study design does not permit precise fragility assessment since calculation requires 1:1 randomization into control and experimental groups (SAVE-MORE randomized patients 2 anakinra:1 control). The apparent non-robust mortality data in SAVE-MORE is not unusual for COVID-19 clinical studies. An overview of COVID-19-related RCTs revealed a median fragility index of 2.5. This means an average mortality change of 2–3 patients would nullify statistical significance of positive study results (Itaya et al., 2022). De-emphasis of mortality as a primary outcome in HCE studies has been specifically criticized in a survey of COVID-19 clinical trials. This survey showed HCEs endpoints predominated and included the comment “when lives are at stake, the trials should literally measure if lives can be saved; any other endpoint would be like a straw in the river- a society drowning in a pandemic may clutch at it with optimism but would nevertheless not be rescued” (Desai and Gyawali, 2020).

Use of dexamethasone in 80–90% of both treatment and placebo groups is a potential concern, since dexamethasone is expected to suppress both IL-1 production and IL-1 bioactivity (Chin and Kostura, 1993; Monick et al., 1994; Jeon et al., 2000). The extremely low circulating levels of IL-1 in COVID-19 (**Step 2d**) poses a conceptual challenge for understanding additive benefit of anakinra when used with inflammation-suppressive corticosteroids. Summarizing intrinsic issues with SAVE-MORE, we note a composite endpoint that is clinically challenging to interpret with outcomes of unequal clinical significance, an anakinra benefit that may not project into the future, and no established mortality benefit. The reported significant mortality reduction in anakinra patients was not a primary endpoint and the reported mortality reduction appears to be fragile.

#### Step 2b: SAVE-MORE extrinsic concern regarding the record of IL-1 blockade to treat COVID-19

There is a history of treating COVID-19 with anakinra that provides context for interpreting the SAVE-MORE results. The record of clinical investigation includes interventional RCTs and observational investigations. An overview of anakinra effect in COVID-19 concluded anakinra has shown mixed results in benefitting COVID-19 patients (Nguyen et al., 2023). Two broad types of investigations used to assess efficacy of anakinra as a COVID-19 therapy include RCTs and observational studies. First and foremost, randomized controlled trials are interventional experiments that randomly assign COVID-19 patients to intervention (anakinra group) or to no intervention (placebo or standard care) group. This design employs external deliberate specific intervention on a proposed cause of disease (excessive IL-1β for the case of anakinra trials) to assess effect on outcome or clinical course of COVID-19. Two recent systematic reviews and meta-analyses including only RCTs in adults hospitalized with COVID-19 evaluated anakinra therapy with study accrual extending to 2022 and no language restriction (Dahms et al., 2023; Shang et al., 2023). These 2 analyses pooled data from the same 5 anakinra RCTs, and the SAVE-MORE study was included in both reports. Considered together, these analyses found no anakinra mortality benefit at 14-, 28-, or 90-day endpoints. Analysis restricted to studies using higher anakinra doses also showed no mortality benefit. The most recent Cochrane Review assessed anakinra effect in COVID-19 by combining RCT data in adult hospitalized patents up to 5 November 2021 (Davidson et al., 2022). Four RCTs including SAVE-MORE satisfied inclusion criteria, and no anakinra effect on 28-day mortality (2 RCTS) or on mortality at ≥60-days (4 RCTs) emerged. Additionally, no anakinra effect was detected for 28-day clinical improvement (3 RCTs) or for clinical improvement at ≥60 days (1 RCT). Certainty of evidence for these outcomes ranged from moderate to very low. The Cochrane report noted an additional 15 COVID-19 registered anakinra trials with results unavailable. This included 3 completed studies with no results reported, 4 terminated studies, and 3 that were not recruiting patients. It seems doubtful the 4 terminated studies were concluded for efficacy, and the 3 studies with no available results may show no anakinra benefit. Unreported data of this kind can impair evaluation of overall treatment effects due to underrepresentation of negative studies. This has been described in detail by Goldacre (2013).

Several meta-analyses that did not limit assessment to RCTs reported on anakinra in COVID-19 patients. These analyses included observational studies as well as RCTs and descriptions of these analyses are summarized in Table 1. Each meta-analysis in Table 1 reported significant anakinra benefit for reducing mortality or need for mechanical ventilation. Most studies assessed were not RCTs and included various kinds of observational (non-interventional) studies. The number of RCTs in each report compared to the total number of studies analyzed was 0/9 (Barkas et al., 2021), 2/7 (Kyriakoulis et al., 2021), 1/9 (Kyriazopoulou et al., 2021a), 0/4 (Pasin et al., 2021), 1/15 (Somagutta et al., 2021), 0/3 (Wang et al., 2021), and 5/24 (Cavalli et al., 2023). In Cavalli et al., analysis restricted to RCTs included in the meta-analysis showed no mortality anakinra benefit (Cavalli et al., 2023). Comparing estimates of anakinra effect reported in RCTs in Dahms *et al*, Shang et al. and Davidson et al. to effects in observational studies in Table 1 meta-analyses shows a disconnect between RCTs and observational studies. There was absence of anakinra benefit in RCTs and positive net effects in observational studies. We believe this difference weakens the case for anakinra use to treat COVID-19, since RCTs (not observational studies) are considered the “gold standard” design to assess therapy efficacy (Schillaci et al., 2013; Fonarow, 2016; Gerstein et al., 2019; Fanaroff et al., 2020; Wallace et al., 2022). The importance of RCTs in medical investigation is underscored by noting all medical treatment is based on manipulation of a cause of disease with the goal of alleviating disease effect or severity. This represents clinical application of the interventionist account of causation in science, as described in Step3 below (Woodward, 2003; Woodward, 2010). The RCT design can approach this goal by using a parallel control group similar in relevant ways to the intervention group except for the intervention. By comparing outcome or effect in the intervention and control groups it can be inferred that difference(s) are due to manipulation of the cause. For the case of anakinra, IL-1 is a proposed cause of COVID-19 and intervening by blocking IL-1 with anakinra should alter COVID-19 by reducing severity.

**Table 1:** Meta-analyses of studies using anakinra to treat COVID-19 that determined significant anakinra benefit.

Meta-analysis	Year	Number of studies (Individuals in study)	Studies analyzed	Patients	Significant results favoring anakinra	Comments
Barkas et al. (2021)	2021	9 (1,119)	6 retrospective cohort 1 prospective open-label 1 open-label Bayesian randomized study 1 open-label with propensity score matched controls	COVID-19 adults hospitalized without intubation	Lower mortality Lower invasive mechanical ventilation (assessment between days 7–28)	No RCT Risk of bias reported as high in 7 studies and as some risk in 2 studies. Lone randomized trial curtailed for futility.
Kyriakoulis et al. (2021)	2021	7 (1,553)	1 RCT (SAVE-MORE) 1 Randomized open-label 5 observational studies with results adjusted for confounders	COVID-19 hospitalized adults	Lower mortality	All observational studies adjusted for confounders. All studies thought to be low risk for bias.
Kyriazopoulou et al. (2021a)	2021	9 (1,185)	8 observational 1 RCT	COVID-19 hospitalized patients	Lower 28-day mortality	Individual patient-level meta-analysis in 6 of 9 studies. Lone randomized trial curtailed for futility.
Pasin et al. (2021)	2021	4 (184)	4 retrospective	COVID-19 hospitalized patients	Lower mortality at longest follow-up	No RCT
Somagutta et al. (2021)	2021	15 (3,530)	1 RCT 5 observational (retrospective cohort) studies 5 case series 4 case reports	COVID-19 and Age > 18	Lower reported mortality (6 evaluable studies) Lower invasive mechanical ventilation (7 evaluable studies)	1 RCT Lone randomized trial curtailed for futility.
Wang et al. (2021)	2022	3 (492)	1 cohort 1 retrospective cohort 1 prospective observational cohort	Hospitalized COVID-19 patients with SOFA ≥2 (“at the edge of sepsis”)	Lower 28-30-day mortality	No RCT
Cavalli et al. (2023)	2023	24 (5,933)	5 RCT (including SAVE-MORE) 10 retrospective 5 prospective 4 prospective with historical controls	COVID-19 adults with hypoxemic respiratory failure	Lower mortality at longest follow-up Lower intubation + mechanical ventilation	No mortality anakinra benefit in analysis restricted to RCT. Overall risk of bias considered high.

Unlike RCTs, observational studies cannot directly test the concept that IL-1 is a cause of COVID-19 since these designs do not permit uncontested assessment of how changing the cause (blocking IL-1) alters the effect (COVID-19). Observational studies comprise several kinds that can include cohort, cross-sectional, and case–control designs (Mann, 2003). Observational studies are prone to two weaknesses referred to as bias and confounding. Bias and confounding compete with a true cause-effect relationship for explaining association between exposure (presumed cause) and outcome (effect). When present, these weaknesses can distort the relationship between an exposure (anakinra) and clinical outcome like mortality or mechanical ventilator requirement. This distortion can manifest as false positive or false negative association, and can alter the magnitude of a real association.^1^ Observational studies cannot establish causality because they cannot ensure the proposed cause or exposure is the only factor being manipulated that influences the effect or outcome. Bias in studies refers to defects in design that are systematic or intrinsic to the study and can result in artifactual associations (Skelly et al., 2012; Schillaci et al., 2013; Fonarow, 2016; Fanaroff et al., 2020). There are >50 kinds of bias and many can be grouped as selection biases or information biases (Tripepi et al., 2010). Selection bias refers to imbalances in important patient characteristics between the intervention (treatment or anakinra) group and non-exposed control group. Since COVID-19 mortality relates to characteristics like age, sex, comorbidities, habitus, and smoking (Dessie and Zewotir, 2021), if a drug like anakinra is given to exposed patients with few of these mortality risks, and the control group contains patients harboring a surplus of patients with these mortality risks, differences in outcome comparing exposed (anakinra) and unexposed controls may be due to elevated pre-existing mortality risk in controls. In such a case, the drug can appear to be effective even if it is not. While observational studies can be designed to reduce selection bias, observational study design features that can produce imbalances in outcome-associated characteristics can be subtle and difficult to account for. As an example, recruiting people who volunteer for intervention can selectively populate the intervention group with healthier people compared to those in the control group (Ganguli et al., 1998). People who volunteer to be intervened upon can be healthier than people who do not volunteer. Observational studies assessing anakinra may give the drug to COVID-19 patients who volunteered for this intervention, resulting in an anakinra group that may have improved outcome due to healthier patients in the anakinra group compared to less healthy patients in the comparator controls. Therefore, lower mortality in the anakinra group may reflect better COVID-19 prognosis in the healthier volunteer population with little or no contribution of anakinra to lower mortality. Selection bias in clinical investigation was specifically examined by comparing 50 RCTs to 56 observational studies that used historical controls that assessed 6 therapies (Sacks et al., 1982). Outcomes (including mortality) for the same therapies were less favorable in historical controls than in RCT controls; statistical adjustments in historical control groups did not equalize outcomes. The upshot is that selection bias in observational studies can irreversibly influence observational studies to favor intervention. *Information biases* refer to how data in observational studies are acquired and interpreted. If patient information is gathered differently in exposed (anakinra) compared to non-exposed (controls), associations between exposure and outcome may reflect imbalance in data collected and not due to exposure to drug. For example, intervention (anakinra) patients may have more complete data entered into the medical record compared to untreated controls. If missing mortality data is more frequent in controls, and missing mortality data is interpreted as death, this could inflate an apparent anakinra mortality benefit (Grimes and Schulz, 2002; Tripepi et al., 2010).

Confounding is a different kind of problem that can affect observational data. Unlike bias, confounding applies to associations in observational studies that are true associations extracted from the study. The problem arises due to interposition of a hidden factor that is associated with both exposure (drug such as anakinra) and outcome like mortality (Skelly et al., 2012).^2^ In its simplest form there is a separate cause or explanation for the outcome that is statistically associated or linked to the exposure. Since the exposure/drug of interest and the hidden factor(s) both affect the outcome, the association between exposure/drug and outcome represents a combined or mixed association between intended exposure (intervention or drug) along with associated confounder(s). As a hypothetical example, if COVID-19 patients given anakinra (exposure) were moved into hospital areas with an increased number of nursing staff (compared to non-exposed COVID-19 patents), an anakinra association with lower mortality may result from increased nursing attention (Haegdorens et al., 2019). Since increased numbers of nurses in hospital is associated with lower patient mortality (Haegdorens et al., 2019), imbalance in number of nurses caring for anakinra-exposed patients (more nurses) compared to fewer nurses caring for unexposed control patients can be an alternative explanation for an apparent anakinra mortality effect. Unlike bias, there exist statistical manipulations that can reduce the interfering effects of confounding (Grimes and Schulz, 2002; Mann, 2003; Skelly et al., 2012; Varga et al., 2023). However, no confounding-mitigating technique can correct confounding factors that are unmeasured or unknown. A significant advantage of randomization in RCTs is that randomization can balance the distribution of confounding factors known and unknown into exposed (intervention) and unexposed control groups. The concern that observational studies can produce faulty associations has been evaluated empirically. In several reports, interventions initially believed true based on observational studies were subsequently overturned by contradicting RCTs and more advanced clinical understanding (Schillaci et al., 2013; Fonarow, 2016; Fanaroff et al., 2020). Of course, RCTs are also imperfect and subject to biases as well (Phillips et al., 2022), and observational and RCT studies often agree (Benson and Hartz, 2000). However, for the case of anakinra intervention in COVID-19, the results from observational studies and RCTs do not agree. We believe the above discussion weighs in favor of believing RCTs more accurately reflect true anakinra effect compared to observational reports. To summarize, we believe RCTs more likely reflect the true relationship between anakinra and clinical outcome compared to observational data. Currently, higher-quality information weighs against anakinra clinical benefit.

Canakinumab is a recombinant human antibody designed to neutralize IL-1β biological action, and it has been assessed as a possible COVID-19 therapy. The same Cochrane meta-analysis of anakinra COVID-19 effects also assessed canakinumab in COVID-19 patients (Davidson et al., 2022). Two RCTs showed no canakinumab effect on 28-day mortality (2 RCTs) and no decrement in mortality at ≥60 days (1 RCT). No 28-day clinical improvement due to canakinumab was observed (2 RCTs). Certainty of evidence ranged from moderate to very low. The first published meta-analysis of canakinumab treatment in COVID-19 reported lower mortality associated with canakinumab use (Ao et al., 2022). This meta-analysis included 6 studies, but only 2 were RCTs. Visual inspection of the included graph shows no significant mortality anakinra benefit in either RCT. Therefore, canakinumab mortality benefit suggested in this analysis is based on questionable source data.

Distilling the above information, we do not find convincing evidence supporting IL-1 blocking strategies to treat COVID-19. Viewed in context, positive clinical benefits reported in SAVE-MORE appear to represent outlier results. Inability of IL-1 suppressing drugs to treat COVID-19 agrees with previously established inability of rhIL-1ra to treat sepsis generally (**Step 2c** below). Since COVID-19 is a variety of sepsis, negative results in COVID-19 described above were expected (Karakike et al., 2021).

#### Step 2c: SAVE-MORE extrinsic concern regarding history of IL-1 blockade as a sepsis treatment

The history of IL-1 blocking agents used to treat sepsis is relevant when considering anakinra as a COVID-19 treatment. Nearly all cases of severe COVID-19 with attendant organ malfunction or death represent cases of sepsis (Kocak Tufan et al., 2021; Vincent, 2021; Herminghaus and Osuchowski, 2022). A systematic review and meta-analysis quantifying sepsis prevalence in hospitalized COVID-19 patients included 151 studies with 218,184 subjects. Sepsis was defined as severe disease satisfying SEPSIS 1 or 2 criteria (Gul et al., 2017), SEPSIS 3 criteria (Singer et al., 2016), or patients with quick SOFA >2 (Singer et al., 2016). This analysis showed 33.3% sepsis prevalence for adults hospitalized without ICU admission, 77.9% prevalence of sepsis in COVID-19 patients in the ICU, and 51.6% for all hospitalized COVID-19 patients (Karakike et al., 2021). Acute respiratory distress syndrome (ARDS) was the most common organ malfunction in ICU patients at 87.5, and 36.4% in the ICU had septic shock. Since severe COVID-19 is a form of severe sepsis and 2 previous well-designed clinical trials failed to demonstrate rhIL-1ra mortality benefit in sepsis patients (Fisher et al., 1994; Opal et al., 1997), it is difficult to understand why anakinra would benefit COVID-19 sepsis patients. Moreover, numerous alternative therapies that reduce cytokine levels or biological function have uniformly failed to treat sepsis. These failures cast additional doubt on the idea that IL-1 inhibition can treat sepsis (Shapiro et al., 2022).

The SAVE-MORE report references a previously described *in vivo* mouse model designed to show plasma from humans with COVID-19 contains factors that generate inflammation in tissues (Renieris et al., 2022). Localized inflammation in mouse tissues was indicated by tissue concentrations of tumor necrosis factor alpha, IL-6, interferon gamma, and myeloperoxidase. Results showed human COVID-19 plasma injected into mice induced compartmented inflammation. These studies also indicated human calprotectin stimulated synthesis of mouse IL-1α, and IL-1α in turn induced the inflammation. Two notable outcomes included correlation between plasma levels of human calprotectin and circulating suPAR concentrations, and tissue inflammation was suppressed in mice treated with anakinra. These results suggested suPAR could serve as a quantitative surrogate marker of inflammation in COVID-19 patients, and the idea that IL-1 blockade could reduce COVID-19-caused tissue-specific inflammation. It appears these observations encouraged use of suPAR as a measure of inflammation in the SAVE-MORE trial, and supported use of anakinra as a COVID-19 treatment. However, given the goal of serving as a model of COVID-19, the conceptual foundation supporting this mouse *in vivo* model likely misrepresents human disease. Although it presents interesting and well-conducted experimental results, the model appears to mirror animal models of sepsis using endotoxin infusion. The failure of animal endotoxin sepsis models to represent natural human sepsis has been described (Piper et al., 1996; Deitch, 1998; Rittirsch et al., 2007). We believe the same kind of disconnect applies to these anakinra mouse studies. There is a special kind of interdependence between these *in vivo* mouse studies and the SAVE-MORE clinical trial that may explain why sepsis research has been steadfastly unproductive as a generator of specific sepsis therapies.

#### Step 2d: SAVE-MORE extrinsic concern regarding low IL-1 levels in COVID-19

Interleukin-1 is produced as 2 forms referred to as IL-1α and IL-1β. Both bind the type 1 IL-1 receptor and are likely biologically equivalent (Di Paolo and Shayakhmetov, 2016). Anakinra blocks biological activity of both IL-1 forms. Most IL-1 sepsis research and clinical investigation has focused on IL-1β, with IL-1α receiving less attention. If anakinra is indeed a treatment for severe COVID-19, it seems IL-1 in blood should be present at amounts sufficient to cause severe disease, organ malfunction, or death. Since no report provides a general assessment of IL-1 concentrations in COVID-19, we examined studies that quantified IL-1 in plasma or serum using enzyme-linked immunosorbent assay (ELISA) or bead-based immunoassay technologies. One large study measured serum IL-1β in 1,715 hospitalized COVID-19 patients that included 1,959 measurements obtained at hospital admission. Median and mean IL-1β concentrations were 0.4 pg./mL and 0.9 pg./mL, respectively, with an upper 99^th^ percentile level of 8.3 pg./mL. No significant difference was noted comparing IL-1β in COVID-19 patients with levels in 9 healthy controls (Del Valle et al., 2020). A second sizable study analyzed plasma IL-1β in 168 COVID-19 patients and revealed a median 1.52 pg./mL. Median IL-1β in 8 healthy controls was 3.0 pg./mL (Mudd et al., 2020). In 118 hospitalized COVID-19 patients (78% considered severe), 0.11 pg./mL median IL-1β was measured in plasma. In 44 healthy controls 0.10 pg./mL was observed with no difference in levels determined between COVID-19 patients and controls (Hawerkamp et al., 2023). Serum IL-1β was measured in 46 COVID-19 patients in intensive care (median 0.35 pg./mL), in 30 COVID-19 patients not in intensive care (median 0.32 pg./mL), and in 24 healthy volunteers (median IL-1β 0.11 pg./mL). No significant IL-1β difference between intensive care and non-intensive care COVID-19 patients was observed (Mazaheri et al., 2022). In serum collected 1–11 days after onset of SARS-CoV-2 infection, median IL-1β in 4 asymptomatic persons infected with SARS-CoV-2 was 6.67 pg./mL, 13.07 pg./mL was measured in 66 symptomatic persons with COVID-19, and in 4 healthy controls median IL-1β was 4.78 pg./mL (Chi et al., 2020). Plasma IL-1β in hospitalized COVID-19 patients in China showed median level of about 1.5 pg./mL in 13 ICU patients and about 1.5 pg./mL in 27 non-ICU patients; 4 healthy control subjects had median levels of approximately 0.3 pg./mL. No sample had a level > 8.0 pg./mL and only 7 values were > 2.0 pg./mL (Huang et al., 2020). It seems IL-1α is studied less frequently in COVID-19 than IL-1β. In the 168 patients studied in Mudd et al. plasma IL-1α median was 2.9 pg./mL and in 8 healthy controls IL-1α median was 2.4 pg./mL (Mudd et al., 2020). To derive perspective on blood IL-1β concentrations in COVID-19, we calculated the weighted average of median IL-1β concentrations in the 2,187 patients described in the studies above. Median values were used since medians were most reported. The weighted average of median blood IL-1β in COVID-19 is 0.88 pg./mL. Measurement of circulating IL-1β concentrations may underestimate or overestimate true concentrations due to factors that interfere with assay performance. As an example, substances like soluble IL-1 receptors may mask recognition of IL-1β in immune recognition-based assays by blocking access of antibody to IL-1β that is required for detection. This would be expected to underestimate true IL-1β concentrations The limited literature on problems that may confront multiplex bead-based assays discusses this phenomenon (Khan et al., 2004; Phillips et al., 2006; de Jager et al., 2009). However, the 3 studies describing most COVID-19 patients referenced above measured IL-1β in 2,001 patients and in healthy controls. These reports showed circulating IL-1β levels no higher in COVID-19 patients compared to levels in uninfected healthy control persons. This suggests IL-1β blood levels are minimally elevated, if at all, in COVID-19 patients independently of assay deficiencies. With assay caveats in mind, IL-1β concentrations in COVID-19 appear to be far lower than levels we determined in patients with sepsis (mean 21.8 pg./mL) (Gharamti et al., 2021). Higher IL-1β amounts in sepsis compared to COVID-19 carries implications. Since Interleukin-1 blockade has proved ineffective as a sepsis treatment (**Step 2c**), it is difficult to understand how blocking IL-1 would prove effective for treating COVID-19 where levels appear to be lower. Based on measurements of IL-1β in the circulation, we conclude IL-1 concentrations seem too low to provide rationale for using IL-1 blockade to treat COVID-19.

It has been conjectured that there is a tissue reservoir of IL-1β that is not reflected by IL-1β levels in blood. Supporting information includes studies in humans with genetic anomalies in the *NLRP3* gene. These anomalies result in gain-of-function overactivation of the IL-1b-activating-NLRP3-associated inflammasome and excessive IL-1β production (Lachmann et al., 2009). These anomalies cause a group of rare human diseases collectively known as cryopyrin-associated periodic syndromes (CAPS) and includes Muckle-Wells Syndrome, Familial Cold Autoinflammatory Syndrome, and Neonatal Onset Multisystem Inflammatory Disease (Kuemmerle-Deschner, 2015; Welzel and Kuemmerle-Deschner, 2021). Although originally characterized as 3 separate diseases, they are now thought to represent different clinical expressions of the same underlying condition with varying severity (Kuemmerle-Deschner, 2015; Welzel and Kuemmerle-Deschner, 2021). Lachmann et al. set out to determine IL-1β kinetics in CAPS patients, which is difficult to determine due to the short IL-1β half-life of 3.5 h. On the other hand, the half-life of inactivated IL-1β complexed to canakinumab (see Step 2b above) increases to 30 days (Lachmann et al., 2009). Lachmann et al. used canakinumab as a trap to maintain IL-1β in blood and in tissues which enabled IL-1β measurement without the complicating factor of rapid IL-1β metabolism. A 2-compartment model (plasma and interstitial fluid) was derived that calculated whole-body daily IL-1β synthesis. The model calculated 6 ng/day whole-body IL-1β production in healthy volunteers and total body IL-1β production of 31 ng/day in CAPS patients. In the absence of canakinumab infusion, serum IL-1β in CAPS patients and in healthy controls was undetectable using an IL-1β assay with detection limit 0.1 pg./mL. Therefore, increased IL-1β synthesis (determined using canakinumab) in CAPS patients was not detectable by measuring IL-1β in blood. Since it is assumed IL-1β synthesis occurs in the tissue compartment, results of this study present the possibility of a hidden IL-1β tissue reservoir in sepsis that is not reflected in IL-1β blood measurements. While there is need for further study, caution should be exercised before assuming existence of a similar hidden tissue reservoir of IL-1β in sepsis like the case for CAPS. First, tissue concentrations of IL-1β in sepsis are unknown and the existence of such a reservoir is unestablished. Second, it is difficult to understand a cause for diffuse IL-1β tissue production in sepsis as is thought to occur in CAPS patients. Genetic anomaly in CAPS likely affects many or all cells capable of producing IL-1β, whereas sepsis usually originates from a focal infection and far less whole-body IL-1β production is expected compared to CAPS patients. Third, the established and often dramatic beneficial effect of IL-1 blocking therapies in CAPS contrasts dramatically with difficulty showing benefit of IL-1 inhibition in sepsis (see Step 2c above) (Kuemmerle-Deschner, 2015; Welzel and Kuemmerle-Deschner, 2021). If a large, sequestered tissue source of IL-1β causes sepsis, it is mysterious why IL-1 inhibiting therapies have not conferred clinical benefit. Third, the clinical course of CAPS contrasts markedly with that in sepsis. Cryopyrin-associated periodic syndromes are not characterized by the acute organ malfunction or death in advanced cases of sepsis. Despite belief there is unrelenting lifelong inflammation in some patients, life expectancy in milder forms of CAPS patients is similar to longevity in the population (Ahmadi et al., 2011). In more severe cases of CAPS mortality up to 20% has been described (Kuemmerle-Deschner, 2015). Although there is no definitive understanding of the cause of death in CAPS patients, it seems reasonable that amyloidosis is a primary etiology (Rodrigues et al., 2022). Amyloid deposition sufficient to cause organ dysfunction of death requires a prolonged period of inflammation and induction of acute-phase proteins. This emphasizes the fact no obvious mechanism links inflammation to the kind of acute disease seen in COVID-19 or sepsis generally.

Is there empirical evidence suggesting existence of an occult IL-1β tissue reservoir that contributes to COVID-19 pathogenesis? For COVID-19, the most relevant conjectured location for tissue IL-1β bioactivity is within lung tissues. Lung tissue constituents may be accessible for study through acquisition of bronchoalveolar lavage fluid (BALF) acquired during bronchoscopy by injected saline into lung segments and then aspirating the fluid along with entrapped contents. Several studies investigating BALF IL-1β levels in patients with COVID-19 have been reported (Nossent et al., 2021; Reynolds et al., 2021; Zaid et al., 2021; Cambier et al., 2022; Voiriot et al., 2022). These reports showed variable median IL-1β BALF concentration between 1.0–1,000 pg./mL. The BALF results in Cambier et al. were different from the other studies with a BALF IL-1β median about 1,000 pg./mL. This contrasts with the 4 other studies reporting median IL-1β BALF concentrations of approximately 1, 3. 60, and 100 pg./mL. To assess the proposal that IL-1β is selectively elevated in a lung tissue reservoir, it is relevant to understand how amounts of IL-1β in BALF compare to levels in the circulation. No conclusive studies in COVID-19 patients address this comparison. A study in 70 mechanically ventilated immunocompetent patients with pneumonia-associated ARDS measured 22 biomarkers (IL-1β was not measured) in both BALF and blood (Bendib et al., 2021). Thirteen of the biomarkers were cytokines, and the ratio of BALF to serum biomarker levels was calculated. Twenty-one of 22 biomarkers studies showed no difference in levels comparing BALF to serum, with only IL-8 showing significant elevation in BALF compared to serum. Although IL-1β may be exceptional and indeed show elevated BALF levels compared to amounts in blood, this is not established. Bendib et al. data do not support existence of an occult substantial tissue cytokine storm in sepsis (Bendib et al., 2021). A full understanding of relative IL-1β levels in BALF compared to blood awaits additional investigation.

Interleukin-1 beta bioactivity in lung tissues is most relevant for assessing a role for localized IL-1β in tissue damage. A study in patients with ARDS or at risk for ARDS examined specific BALF IL-1 biological activity by measuring IL-1β effect on A549 cell adhesion molecule response (an IL-1 biological action) in samples collected over 21 days (Park et al., 2001). This communication showed significantly increased BALF IL-1β bioactivity in BALF (compared to healthy controls) only at day 1 of study in patients with ARDS or at risk for ARDS. No increased IL-1β bioactivity was observed over the remaining 21 days of study. No significant BALF difference in IL-1β protein quantification in patients compared to healthy controls was observed over the entire 21 days of assessment. Simultaneous BALF measurement of IL-1β and the natural IL-1 antagonists IL-1ra and soluble IL-1 receptor type 2 (sIL-1Rll) showed substantial excess of the IL-1β antagonists compared to IL-1β. Molar ratios of IL-1ra to IL-1β in BALF ranged between 125-fold to 500-fold, and the molar ratios for sIL-1RII to IL-1β ranged from 2-fold to 200-fold. There was statistically significant molar ratio overabundance of antagonist compared to IL-1β for one or both antagonists for days 1–7 in ARDS patients compared to controls. This exuberant production of IL-1 inhibitors may account for the near absence of IL-1β biological activity in BALF in these patients. Concluding this Step, it seems likely there are very low IL-1β levels in the circulation in COVID-19 patients, and levels are likely lower than average levels in sepsis patients. Studies of BALF IL-1 weigh against the presence of an occult substantial tissue reservoir of bioactive IL-1. The idea there is compartmentalized IL-1β in tissues sufficient to cause organ failure or death is unestablished. Available data, though not definitive, seem inauspicious for this concept.

#### Step 2e: SAVE-MORE extrinsic concern regarding how much IL-1 is present in sepsis and how much IL-1 humans can tolerate

Detailed understanding of IL-1 concentrations in sepsis has received little attention. Relatedly, there is little understanding of amounts of IL-1 humans can tolerate without severe clinical consequences. Answers to both questions seem important for understanding the conceptual rationale for treating COVID-19 (or sepsis in general) with IL-1 blocking agents. In response, we conducted a systematic review and meta-analysis that quantified IL-1 levels in sepsis and determined a mean 21.8 pg./mL IL-1β in the circulation in sepsis patients (Gharamti et al., 2022), demonstrating IL-1β blood levels in sepsis far exceed those in COVID-19 (**Step 2d**). Since blocking IL-1 with IL-1ra has failed as a sepsis therapy (Fisher et al., 1994; Opal et al., 1997), it is difficult to understand how blocking IL-1 would benefit COVID-19 patients but not sepsis patients.

The importance of understanding how much IL-1β can be tolerated by humans is underappreciated. The claim that IL-1 in the circulation causes sepsis depends on the presence of IL-1 amounts sufficient to generate severe disease or death. There are obvious ethical restrictions for determining how much IL-1 is needed to harm humans. However, there exists a small but relevant history of IL-1 infusion into humans that provides clues to how much IL-1 humans can tolerate. Recombinant IL-1β was infused intravenously over 30 min into 19 human cancer patients at several doses that produced calculated serum concentrations between 50–2,550 pg./mL (Crown et al., 1991). Interleukin-1β was well-tolerated with non-pressor-requiring hypotension being the most serious adverse effect and 2 patient withdrawals due to hypotension at the highest dose (calculated IL-1β of 2,550 pg./mL). Several clinical reports describe intravenous infusion of IL-1α in cancer patients. Since cell signaling is equivalent for IL-1α and IL-1β, they are predicted to have similar physiological effects (Di Paolo and Shayakhmetov, 2016). Intravenous IL-1α was infused over 15 min in 15 cancer patients daily for 7 days with each dose producing calculated serum levels between 255 and 25,450 pg./mL (Smith et al., 1992). Calculated concentrations below 2,545 pg./mL were well-tolerated, and calculated concentrations 7,635 or 25,450 pg./mL produced occasional hypotension requiring volume or pressor support. In a separate report in 10–15 cancer patients, IL-1α was infused intravenously over 15 min daily for 5 days at doses producing calculated serum concentrations 760–7,600 pg./mL (Smith et al., 1993). This study assessed IL-1α as a drug to bolster platelet levels in cancer patents. Calculated IL-1α levels up to 2,500 mg/mL were well-tolerated. Patents receiving the highest dose and calculated serum concentrations of 7,600 pg./mL had reversible hypotension in 9/15 patients and mild pulmonary capillary suffusion in 6 of 15 patients. These analyses of IL-1 tolerability have several shortcomings, including serum IL-1 concentrations that were calculated based on body mass and blood volume estimates and may inaccurately represent true circulating IL-1 levels. Also, adverse IL-1 effects described in these studies likely overestimate detrimental IL-1 effects compared to effects in natural sepsis. In these studies, IL-1 was infused intravenously as a bolus given over minutes. Several studies injected IL-1 repeatedly over several days. Natural infection will produce more gradual cytokine elevations (unlike a bolus infusion), and repeated IL-1 surges caused by recurring injections does not have a natural counterpart. Importantly, natural sepsis is accompanied by substantial increases in circulating endogenous IL-1 suppressors IL-1ra and sIL-1Rll (Waage and Steinshamn, 1993; van der Poll et al., 1997; Olszyna et al., 1998). In contrast, bolus IL-1 infusions elevate IL-1 unaccompanied by increased natural IL-1 inhibitors. Consequently, biologically active IL-1 during sepsis will be lower than measured IL-1 levels. It is also likely that many natural sepsis patients possess increased physiological reserve and may tolerate IL-1 adverse effects better than IL-1-infused late-stage cancer patients. With caveats, we conclude there is substantial conceptual distance between IL-1 levels that produce organ malfunction or death and concentrations observed in COVID-19 or sepsis patients. The magnitude of difference between IL-1β levels observed in COVID-19 (about 1.0 pg./mL see **Step 2d**) or sepsis (approximately 20 pg./mL (Gharamti et al., 2021)) compared to IL-1 amounts capable of causing severe disease in humans appears to span orders of magnitude. It appears humans tolerate IL-1 levels of 2,500 pg./mL, and levels of about 7,600 pg./mL can be tolerated with non-severe adverse effects. We believe the preponderance of evidence suggests IL-1β is not a cause of organ malfunction or death in COVID-19.

### Step 3– is there a good reason to believe hyperinflammation is a cause of sepsis?

Due to **Step 2** concerns, we view interest in using anakinra to treat COVID-19 as the latest example of an approach to sepsis therapy that is difficult to understand. Problems described in **Steps 2b-e** have the effect of lowering the pre-test probability for success using anakinra to treat COVID-19. Moreover, the underlying concept that supplies rationale to use interventions like anakinra to treat sepsis is defective in several ways. This confers further doubt that anakinra can treat COVID-19. The underlying concept we refer to is the idea that hyperinflammation or cytokine storm is a cause of organ malfunction of death in sepsis. Despite lack of success in developing a specific sepsis therapy using this concept of pathogenesis, the hyperinflammation or cytokine storm idea continues to dominate thinking in sepsis investigation. Viewed within the context of historical record, beneficial use of inflammation-suppressing therapies like anakinra to treat COVID-19 would be a highly improbable event. We published a detailed analysis that shows why the idea that hyperinflammation or cytokine storm causes sepsis is dubious (Shapiro et al., 2022). The hyperinflammation pathogenesis concept of sepsis has not been precisely described. A sketch of what this idea appears to entail is listed as a sequence:

Infectious agents are recognized by host pattern recognition receptor molecules that initiate production of pro-inflammatory substances that include cytokines. Physiological (beneficial) cytokine functions include host immunity enhancement and tissue repair.
Excessive cytokine production sometimes occurs and causes hyperinflammation. This is the “cytokine storm.”
Hyperinflammation causes physiological and structural derangements that can result in organ failure and possibly death.

This duality of cytokine function as both promoters of innate immunity and causes of tissue damage accounts for the proposed “two-edged sword” role for cytokines (Chaudhry et al., 2013). Table 2 summarizes challenges that weaken the case for assigning hyperinflammation as a cause of COVID-19 and sepsis generally (Karakike et al., 2021). The conceptual confusion row in Table 2 refers to ideas or definitions that are vague and imprecise which confounds attempts to apply the hyperinflammation concept to sepsis investigation and treatment. Inflammation itself is the most problematic concept (Kushner, 1998; Weissmann, 2010; Groopman, 2015; Antonelli and Kushner, 2017; Shapiro et al., 2022). No account of inflammation provides criteria that can reliably differentiate pro-inflammatory from anti-inflammatory molecules or categorize molecules into those that cause inflammation from others that are non-causal effects or markers of inflammation. This imprecision, combined with the advent of multiplex assay platforms that can simultaneously measure up to 100 molecules in blood (Kupcova Skalnikova et al., 2020) has resulted in conflicting reports enumerating molecules relevant for linking sepsis to inflammation. A sampling of COVID-19 reports list blood levels for 20 (Hawerkamp et al., 2023), 27 (Huang et al., 2020), 35 (Mudd et al., 2020), 48 (Tjan et al., 2021), or 53 (Herr et al., 2021) molecules. These quantifications include molecules thought to be pro-inflammatory like IL-1 and TNF, anti-inflammatory cytokines such as IL-6 and IL-10, interferons, chemokines, soluble cell receptors, complement components, ferritin, tumor markers, the acute-phase protein CRP, procalcitonin, and chitinase 3-like 1 (YKL-40). It is uncertain which of these substances is/are causing COVID-19-associated inflammation as opposed to substances related to but do not participate in COVID-19 inflammation. Assuming hyperinflammation causes sepsis, it is essential to separate molecules that cause inflammation from those that do not. Only those that cause sepsis are candidates for blockade to treat patients. Moving forward seems to necessitate a detailed and precise concept of inflammation that focuses research and clinical study on molecules that truly cause inflammation. Other investigators have called attention to some of these deficiencies (Sinha et al., 2020; Stolarski et al., 2021). The notion of a cytokine storm is also problematic. Supposedly, cytokine storm refers to a suite of molecules serving as messengers/mediators of inflammation that in sufficient quantities cause pathologies that can result in organ malfunction or death (Mayr et al., 2014; Gul et al., 2017; Fajgenbaum and June, 2020). A precise account of what constitutes cytokine storm depends on an understanding of inflammation. Researchers and clinicians seem left to decide for themselves which molecules in what combinations are elevated to unspecified amounts constitutes a genuine “storm.” Therefore, the conceptual confusion intrinsic to our ideas about inflammation apply equally to cytokine storm. Given imprecision of the terms inflammation and cytokine storm, it is unsurprising there is no characterization of amounts of inflammatory molecules that portend transition from a useful pathogen-defeating inflammatory response into a destructive hyperinflammation condition. This quantitative uncertainty questions the usefulness of the “2-edged sword” analogy, where inflammation is useful when present at some appropriate amount and detrimental when present at undefined excessive levels (Chaudhry et al., 2013). Since sepsis is described as a “dysregulated host response to infection,” the conceptual foundation of sepsis appears to depend on understanding inflammation (Singer et al., 2016). We believe it is essential to develop notions of inflammation, cytokine storm, and sepsis that can be used to direct and constrain investigation in a way that permits us to test the hyperinflammation sepsis idea. Improved accounts can guide conduct of better-defined experiments that can be used to qualify results as confirmatory or disconfirmatory for the hyperinflammation idea. This is pointed out in the last column of the first row in Table 2. As things stand now, it appears no conceivable experimentation can falsify or discredit the hyperinflammation concept. More precise concepts may narrow the scope of acceptable experimental conduct and qualify results into those that count as confirmatory data.

**Table 2:** Does hyperinflammation cause sepsis?

Defect category	Specific defects	Comments and consequences	Proposed solution
Conceptual confusion	Inflammation. Cytokine storm. Sepsis. 2-edged sword.	Poorly defined terms promote inconsistent and haphazard basic and clinical investigation.	Construct novel concepts that are precise, scientifically useful, and capture intuitive understanding of these terms. This can help direct and focus future investigation.
Causality (hyperinflammation as a putative cause of sepsis)	Interventionist account of causality not satisfied (gold standard) (Woodward, 2003).	Hyperinflammation cannot be upheld as a cause of sepsis.	Create an alternative account of sepsis that satisfies this criterion of causality.
Mathematics	Cytokine concentrations in sepsis too low to cause organ malfunction or death. Humans tolerate cytokine levels far higher than levels reported in sepsis.	Underappreciation of significance of available quantitative data.	Follow the numbers. Conduct analyses that compare cytokine levels in sepsis, COVID-19, other diseases. Revisit older literature that helps us understand the limits of human tolerance for elevated cytokines.
History	Repeated failures of anti-inflammation therapies designed to treat sepsis.	Hyperinflammation or cytokine storm is unfalsifiable by experimentation.	Create an alternative account (theory) of sepsis with superior empirical power*.

*Can explain and predict sepsis observations and can be used to control (treat) sepsis.

Reference to causality in Table 2 points out hyperinflammation does not satisfy the essential criterion that can identify inflammation as a cause of sepsis. At the heart of experimental evidence showing causality is understanding what a phenomenon (like sepsis) depends on. This notion is captured by an interventionist account (“gold standard”) of causation (Woodward, 2003; Woodward, 2010). Intuitively, if you wiggle a genuine cause, you should jiggle the effect. Manipulating the cause changes the effect. Applying this interventionist account of causality to the idea that hyperinflammation causes sepsis, it should be the case that intervening (blocking) inflammation should alter/treat/cure sepsis. Since numerous studies have failed to treat sepsis by blocking inflammation, the hyperinflammation concept of sepsis has not satisfied the gold standard criterion for causation. The final column in row 2 of Table 2 points out an alternative concept of sepsis should fulfill the interventionist criterion of causation. Mathematical challenges in Table 2 refers to the importance of focusing attention on accurate quantification of relevant cytokines in sepsis and comparing these values to those in other diseases. Are cytokine levels in severe sepsis truly extraordinary, or are they comparable to amounts seen in diseases with far less or minimal severity? We responded to this need with our report on these issues (Gharamti et al., 2022), but more work needs to be done. A related mathematical consideration is an understanding of how much cytokine humans can tolerate. This can help us understand cytokine levels measured in sepsis by answering the question “is that a lot?” This is discussed for IL-1β in Step 2e above and for tumor necrosis factor alpha in a prior report (Shapiro et al., 2022). It is fair to say humans can tolerate amounts of cytokines far more than what is intuitively believed. Finally, the role of history in the hyperinflammation characterization of sepsis refers to the peculiar fact the hyperinflammation concept persists without question despite prodigious failure in clinical trials. The hyperinflammation concept of sepsis appears to be unfalsifiable. This refers to remarkable persistence of the hyperinflammation concept despite a striking history of unsuccessful clinical trials based on this concept. We think survival of the hyperinflammation idea in the face of this record is a fascinating and understudied phenomenon. This kind of falsification-resistance in science has been the subject of intense study and discussion (Kuhn, 1962; Popper, 1972). As we have described elsewhere, characterizing hyperinflammation as a scientific paradigm in the sense intended by Thomas Kuhn may account for resistance to falsification (Kuhn, 1962; Wray, 2011; Shapiro et al., 2022). We believe the only cure for this problem is to generate a competing sepsis theory that possesses more empirical power than the hyperinflammation concept. Empirical power of a novel sepsis theory refers to three pivotal features. These features include first a way to explain sepsis which amounts to showing what sepsis depends on. This would include a description of cause-effect interactions that combines objects or cell functions within the theory (examples may include specific cytokines or other molecules, defined cell activities) in a way that produces clinical manifestations of sepsis (Woodward, 2003; Woodward, 2010; Illari et al., 2011). The second feature is the capacity to make predictions about yet unobserved empirical (including clinical) facts or experiments (Carnap, 1946). Finally, an effective theory would point the way to effective sepsis interventions (treatments). In this final sense, the practice of medicine is like engineering due to the existence of a bottom line or goal (treatment of patients) and appropriation of scientific concepts from other fields that can be applied to this bottom line (Illari et al., 2011). Comparing empirical power of competing theories can provide criteria for recognizing a superior concept of sepsis. In our view, the most promising path to progress in understanding and treating sepsis resides in the realm of novel theory development (Shou et al., 2015). Empirical disconfirmations of the current hyperinflammation concept have failed to propel us forward, and we see little reason to expect advancement in the future.

### Step 4– cycles of sepsis futility

Decades of testing sepsis therapies in patients has been characterized by cycles of repetitive negative clinical trials punctuated by intermittent positive studies. This has been followed invariably by loss of enthusiasm for these positive results (Quezado et al., 1994; For sepsis, the drugs don't work, 2012) Prominent examples of this pattern include initial excitement about HA-1A, a human monoclonal antibody directed against endotoxin to treat sepsis. This antibody preparation was approved for clinical use in Europe and in parts of Asia but not in the United States (Sweeney et al., 2008). Approval for clinical use in Europe was withdrawn 1993 after follow-up studies showed lack of mortality benefit (perhaps harm) in sepsis patients. Similarly, human activated protein C showed initial promise as an adjunctive sepsis therapy with marketing approval for sepsis in 2001. Once again, follow-up data suggested absence of clinical sepsis benefit and the drug was withdrawn in 2011. Use of a cocktail of hydrocortisone, vitamin C, and thiamine to treat sepsis was widely publicized as an antisepsis therapy that lowered sepsis mortality (Marik et al., 2017). However, this approach suffered enthusiasm decay following a series of nonconfirmatory clinical studies (Chang et al., 2020; Fujii et al., 2020; Mitchell et al., 2020; Mohamed et al., 2020; Moskowitz et al., 2020; Sevransky et al., 2021). This recurring pattern has been described previously (Sweeney et al., 2008). Now we have anakinra to treat the COVID-19 variety of sepsis. Since specific sepsis therapies have never proved conclusively efficacious (**Step 3**), simple induction suggests anakinra COVID-19 therapy will likewise fail to become an established COVID-19 treatment.

We believe emergence of anakinra as a COVID-19 treatment should be taken as a time to question why we again retrace a pattern of events that will likely conclude with yet another failed attempt to treat sepsis by blocking some element of inflammation. Experience suggests further basic and clinical experimentation is an unlikely means of uncovering successful adjunctive sepsis therapies. We believe a plan for escaping the futility cycle should include 2 parts. First, there should be an explanation for why this cycle exists and recurs. Second, this understanding should be used to generate approaches to avoid these cycles. A quote attributed to Einstein states insanity is doing the same thing over and over and expecting different results. This reminds us that we should recognize the existence of repeated cycles of failure and take steps to avoid repetitions. Sepsis investigation is caught in this kind of cycle. An often-overlooked necessity to follow Einstein’s advice is the need to understand what “the same thing” refers to. Correcting mistakes requires knowing why we make them.

After publication of the SAVE-MORE results, a randomized controlled open-label phase 2/3 trial assessed intravenous anakinra in COVID-19 patients with severe pneumonia (Fanlo et al., 2023). Enrollees were adults (age ≥ 18) with PCR-proved SARS-CoV-2 infection with pneumonia (infiltrates in lung imaging), and presumed elevated inflammation indicated by IL-6 > 40 pg./mL, ferritin >500 ng/mL, C-reactive protein >3 mg/dL, or lactate dehydrogenase >300 U/L. Severe pneumonia was required and defined by room air pulse oximetry percent ≤94, oxygen pressure/fraction inspired oxygen ≤300, or pulse oximetry percent/fraction inspired oxygen ≤350. Control patients received standard of care and the anakinra group received standard care with intravenous anakinra 100 mg 4 times daily for up to 15 days. Randomization was 1:1 and primary outcome was absence of mechanical ventilation assistance at 15 days after study entry. Mechanical ventilation could be either invasive (endotracheal intubation with ventilator support) or non-invasive (presumedly use of mechanical ventilator device without intubation). Several secondary outcomes were assessed that included 28-day mortality and viral clearance from nasopharyngeal swab specimens by day 15. The intention-to-treat analysis included 78 control patients and 83 anakinra patients. Methylprednisolone was given to controls (39.1%) and to anakinra patients (37.1%) as part of standard care. The primary outcome of absent mechanical ventilation use up to day 15 was not significantly affected by anakinra (77.1% in anakinra vs. 85.9% control with relative risk = 0.9 and *p* = 0.16). In fact, anakinra use associated with increased use of mechanical ventilation up to day 15, albeit without statistical significance. Selected secondary endpoint analyses showed no anakinra effect on 28-day mortality and no effect on viral load clearance by day 15. Does this study indicate we are now entering the kind of cycle described above?

## Conclusion

Recent government support to use anakinra to treat COVID-19 should remind us of the unproductive history of developing specific sepsis therapies. There are none. We believe caution should accompany interpretation of the positive SAVE-MORE results and we recommend reflection before adoption. Some intrinsic design features of SAVE-MORE temper persuasiveness to use anakinra as a COVID-19 treatment. Taking a general view, we believe SAVE-MORE should be interpreted in the context of previous sepsis and COVID-19 investigation. Pooled analyses of RCTs testing anakinra use in COVID-19 have not shown benefit, and analysis of IL-1 concentrations in COVID-19 and in sepsis generally are inconsistent with a significant role for IL-1 in pathogenesis. Clinical study of antiinflammation strategies to treat sepsis has been characterized by a predictable cycle of abundant clinical failures punctuated by an intermittent positive result. Subsequently, the positive result fades from prominence. We fear anakinra use to treat COVID-19 is already tracing this familiar path (Fanlo et al., 2023), and we may find ourselves back where we started. We believe any pathway to successful sepsis therapy will require escape from this recurring pattern. There is need to understand the larger forces at work that compel us to use these kinds of therapies to treat this kind of disease.

## Data availability statement

Publicly available datasets were analyzed in this study. This data can be found here: Del Valle, D.M., Kim-Schulze, S., Huang, H.H., Beckmann, N.D., Nirenberg, S., Wang, B., et al. (2020). An inflammatory cytokine signature predicts COVID-19 severity and survival. Nat Med 26(10), 1,636–1,643. doi: 10.1038/s41591-020- 1051-9. Mudd, P.A., Crawford, J.C., Turner, J.S., Souquette, A., Reynolds, D., Bender, D., et al. (2020). Distinct inflammatory profiles distinguish COVID-19 from influenza with limited contributions from cytokine storm. Sci Adv 6(50). doi:10.1126/sciadv.abe3024. Hawerkamp, H. C., Dyer, A. H., Patil, N. D., McElheron, M., O’Dowd, N., O’Doherty, L., et al. (2023). Characterisation of the pro-inflammatory cytokine signature in severe COVID-19. Front. Immunol. 14:1170012. doi: 10.3389/fimmu.2023.1170012. Mazaheri, T., Ranasinghe, R., Al-Hasani, W., Luxton, J., Kearney, J., Manning, A., et al. (2022). A cytokine panel and procalcitonin in COVID-19, a comparison between intensive care and non-intensive care patients. PLoS One 17(5), e0266652. doi:10.1371/journal. pone.0266652. Chi, Y., Ge, Y., Wu, B., Zhang, W., Wu, T., Wen, T., et al. (2020). Serum Cytokine and Chemokine Profile in Relation to the Severity of Coronavirus Disease 2019 in China. J Infect Dis 222(5), 746–754. doi:10.1093/infdis/jiaa363. Huang, C., Wang, Y., Li, X., Ren, L., Zhao, J., Hu, Y., et al. (2020). Clinical features of patients infected with 2019 novel coronavirus in Wuhan, China. Lancet 395(10223), 497–506. doi:10.1016/S0140-6736(20)30183-5.

## Author contributions

LS conceived general ideas and wrote the initial draft. SS, CF-P, AG, and AH-M contributed to concepts expressed in the manuscript, contributed to writing the manuscript, and reviewed and corrected the text. All authors contributed to the article and approved the submitted version.

## Footnotes

^1^https://www.healthknowledge.org.uk/public-health-textbook/research-methods/1a-epidemiology/biases

^2^https://quantifyinghealth.com/confounding-vs-bias/

## Abbreviations

CAPS: Cryopyrin-Associated Periodic Syndromes
CDC: Centers for Disease Control and Prevention
COVID: Coronavirus Disease 2019
EMA: European Medicines Agency
EUA: Emergency Use Authorization
FDA: Food and Drug Administration (USA)
HCE: Hierarchical Composite Endpoint
IL: Interleukin
ICU: Intensive care unit
PCR: Polymerase-Chain Reaction
rhIL-1ra: recombinant human IL-1 receptor antagonist
RCT: Randomized Controlled Trial
SARS-CoV-2: Severe Acute Respiratory Syndrome Coronavirus 2
SAVE-MORE: suPAR-guided Anakinra treatment for Validation of the risk and early Management OF seveRE respiratory failure by COVID-19
SIRS: Systemic Inflammatory Response Syndrome
SOFA: Sequential Organ Failure Assessment
sIL-1Rll: soluble IL-1 receptor type 2
suPAR: soluble urokinase Plasminogen Activator Receptor
WHO: World Health Organization
WHO-CPS: WHO Clinical Progression Scale
